# A Novel Tiller Angle Gene, *TAC3*, together with *TAC1* and *D2* Largely Determine the Natural Variation of Tiller Angle in Rice Cultivars

**DOI:** 10.1371/journal.pgen.1006412

**Published:** 2016-11-04

**Authors:** Haijiao Dong, Hu Zhao, Weibo Xie, Zhongmin Han, Guangwei Li, Wen Yao, Xufeng Bai, Yong Hu, Zilong Guo, Kai Lu, Lin Yang, Yongzhong Xing

**Affiliations:** 1 National Key Laboratory of Crop Genetic Improvement and National Center of Plant Gene Research (Wuhan), Huazhong Agricultural University, China; 2 Hubei Collaborative Innovation Center for Grain Industry, China; University of Minnesota, UNITED STATES

## Abstract

Tiller angle is one of the most important components of the ideal plant architecture that can greatly enhance rice grain yield. Understanding the genetic basis of tiller angle and mining favorable alleles will be helpful for breeding new plant-type varieties. Here, we performed genome-wide association studies (GWAS) to identify genes controlling tiller angle using 529 diverse accessions of *Oryza sativa* including 295 *indica* and 156 *japonica* accessions in two environments. We identified 7 common quantitative trait loci (QTLs), including the previously reported major gene *Tiller Angle Control 1* (*TAC1*), in the two environments, 10 and 13 unique QTLs in Hainan and Wuhan, respectively. More QTLs were identified in *indica* than in *japonica*, and three major QTLs (*qTA3*, *qTA1b*/*DWARF2* (*D2*) and *qTA9c*/*TAC1*) were fixed in *japonica* but segregating in *indica*, which explained the wider variation observed in *indica* compared with that in *japonica*. No common QTLs were identified between the *indica* and *japonica* subpopulations. Mutant analysis for the candidate gene of *qTA3* on chromosome 3 indicated a novel gene, *Tiller Angle Control 3* (*TAC3*), encoding a conserved hypothetical protein controlling tiller angle. *TAC3* is preferentially expressed in the tiller base. The *ebisu dwarf* (*d2*) mutant exhibited a decreased tiller angle, in addition to its previously described abnormal phenotype. A nucleotide diversity analysis revealed that *TAC3*, *D2* and *TAC1* have been subjected to selection during *japonica* domestication. A haplotype analysis identified favorable alleles of *TAC3*, *D2* and *TAC1*, which may be used for breeding plants with an ideal architecture. In conclusion, there is a diverse genetic basis for tiller angle between the two subpopulations, and it is the novel gene *TAC3* together with *TAC1*, *D2*, and other newly identified genes in this study that controls tiller angle in rice cultivars.

## Author Summary

Tiller angle is the key component of plant architecture that greatly affect grain yield. However, few tiller angle-related genes that can be used for improving rice plant architecture have been isolated based on natural variation. Here, we identified 7 common tiller angle-related QTLs by a genome-wide association study, including the previously reported major gene *TAC1*, in two environments in the 529 diverse rice accessions and dozens of QTLs specially identified in one environment. Two QTLs were validated by mutant analysis: A novel gene *TAC3*, encoding a conserved hypothetical protein and preferentially expressing in the tiller base, was the candidate gene of *qTA3*; *d2* mutant exhibited a decreased tiller angle, in addition to its previously described abnormal phenotype. A haplotype analysis identified favorable alleles of *TAC3*, *D2* and *TAC1* in *indica*, which may be used for breeding plants with an ideal architecture, while they were all subjected to selection and fixed in *japonica*. In conclusion, there is a diverse genetic basis for tiller angle between the two subpopulations, and it is the novel gene *TAC3*, together with *TAC1* and *D2* that greatly controls tiller angle in rice cultivars.

## Introduction

As a key factor in rice plant architecture, tiller angle determines the planting density per unit area and contributes greatly to grain yield. The tiller angle is defined as the angle between the main culm and the side tillers [1]. A favorable rice tiller angle is an important component of the ideal plant architecture that has been selected by humans in the long history of domestication and genetic improvement [2]. *Oryza rufipogon*, the progenitor of cultivated rice (*Oryza sativa*), shows a spread-out growth pattern that allows it to escape some diseases induced by high humidity, but this pattern occupies too much space and increases shading and lodging, thus decreasing the photosynthetic efficiency and grain yield per unit area accordingly. By contrast, cultivated rice usually exhibits a better plant architecture, with a smaller tiller angle that leads to high potential yields.

Tiller angle changes dynamically throughout the life cycle of rice to achieve efficient resource use. At tillering stage, tiller angle increases, which enables young plant to occupy a large empty space for subsequent tiller development and simultaneously inhibits weed growth. At later stages, tiller angle decreases, especially at heading stage, when it reaches its minimum, allowing the mature plant to reduce leaf shade and increase its photosynthetic efficiency. Heading stage is a key developmental period in rice, and the planting density per unit area is mainly determined by the tiller angle at this stage.

Due to its importance in rice production, increasing attention has been paid to dissecting the genetic basis of tiller angle over recent decades. Dozens of tiller angle-related quantitative trait loci (QTLs) have been explored in rice via classical bi-parental cross mapping [3–8]. However, most of these QTLs are seldom used for marker-aided selection, due mainly to their minor effects. Recently, a few genes controlling tiller angle have been cloned. *PROSTRATE GROWTH 1* (*PROG1*) has been accepted as a domestication-related gene that controls tiller angle and tiller number during both tillering and heading stages in wild rice. *PROG1* encodes a zinc finger nuclear transcription factor and is located on chromosome 7. Amino acid changes in the PROG1 protein and regulatory changes during domestication led to loss of the function of this gene, which promoted the transition from the prostrate growth tillers of wild rice to erect growth tiller of domesticated rice [9,10]. *Tiller Angle Control 1* (*TAC1*) is a major QTL located on chromosome 9 that controls tiller angle during heading stage in cultivated rice and encodes an expressed protein without homologous genes in rice. A mutation in the 3’-splicing site of the 1.5-kb intron ‘GGGA’, which exists in 88 compact *japonica* rice accessions, decreases the level of *tac1* and leads to a smaller tiller angle; ‘AGGA’ is present in 21 wild rice and 43 *indica* rice accessions with spread-out tillers [11].

Rice shoot gravitropism is suggested to be a key factor affecting plant architecture. The rice *la* mutant, which exhibits a wider tiller angle, has been intensively studied for decades [12–17]. However, the *LAZY1* (*LA1*) gene was not identified until 2007. *LA1* encodes a novel, grass-specific protein that controls shoot gravitropism by regulating polar auxin transport (PAT) [18,19]. *Loose Plant Architecture 1* (*LPA1*) is an INDETERMINATE DOMAIN protein involved in shoot gravitropism that regulates both tiller angle and leaf angle in rice during the vegetative and reproductive stages [20]. Suppression of *OsPIN1* or over-expression of *OsPIN2* (two auxin efflux transporters) alters PAT and increases tiller angle [21,22]. Recent research has demonstrated that strigalactones (SLs), a group of newly identified plant hormones, inhibit auxin biosynthesis and attenuate rice shoot gravitropism primarily by decreasing local indoleacetic acid contents. Multiple *SOLs* (suppressors of *lazy1*) involved in SL biosynthesis, such as *dwarf 17* (*d17*), *d10* and *d27*, or in SL signaling pathways, such as *d14* and *d3*, can rescue the spreading phenotype of *lazy1* [23]. Suppressing the expression of *OsLIC1* (*Oryza* sativa leaf and tiller angle increased controller), a novel CCCH-type zinc finger gene, increases leaf and tiller angles via the BR signaling pathway [24].

Although the identification of these tiller angle-related genes is helpful for understanding the mechanism of tiller angle formation, few genes that can be used for improving rice plant architecture have been isolated based on natural variation. Therefore, mapping additional genes that contribute to the natural variation of tiller angle is required for breeding varieties with an ideal plant architecture resulting in high grain yields. Genome-wide association studies (GWAS) offer a powerful approach to establishing the relationship between DNA markers and phenotypic traits in crops [25]. With the assessment of millions of single nucleotide polymorphisms (SNPs), GWAS can take full advantage of ancient recombination events to identify the genetic loci underlying complex traits at a high resolution using a large number of crop varieties [26]. Many QTLs for agronomic traits have been identified in cultivated rice through GWAS [27–29]. Recently, several novel loci and candidate genes for tiller angle at tillering stage have been identified by GWAS and the elite alleles have been explored for plant architecture improvement [30].

Here, we investigated the tiller angle of 529 *O*. *sativa* accessions at heading stage and performed GWAS separately in the full population and the *indica* and *japonica* subpopulations. We isolated a novel gene, *TAC3*, as well as several novel QTLs in this study. We also identified distinct genetic regulatory mechanisms for tiller angle between the two subpopulations, providing information on how to improve tiller angle in *indica* and *japonica* rice.

## Results

### Phenotypic variation of the tiller angle in rice during heading stage

**Fig 1:**
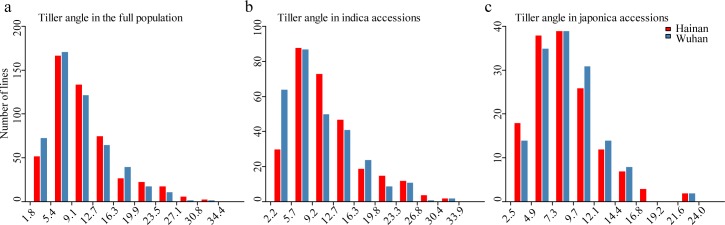
Phenotypic distribution of rice tiller angle. Histogram showing distributions of tiller angle in the full population (a), in *indica* accessions (b) and in *japonica* accessions (c) in Hainan and Wuhan, respectively.

The worldwide rice collection exhibited a distinctive population structure and was classified into nine subpopulations: *indI*, *indII*, *indica* intermediate, *Tej*, *Trj*, *japonica* intermediate, *Aus*, *VI*, and intermediate [31]. Of these 529 accessions, 295 were classified into the *indica* subpopulation, including *indI*, *indII* and *indica* intermediate, and 156 were classified into the *japonica* subpopulation, including *Tej*, *Trj* and *japonica* intermediate. There were large variations in tiller angle throughout the population in both environments. The tiller angle ranged from 2.5° to 34.4° in Hainan and from 1.8° to 31.5° in Wuhan. The largest number of accessions fell into a small range of tiller angles, from 2° to 16° (Fig 1a). The variations observed in the two environments showed a similar distribution, skewed towards smaller tiller angles. A significant correlation was observed between the two environments in the whole population (r = 0.66). However, there was a significant difference in tiller angle between the *indica* and *japonica* subpopulations (Fig 1b and 1c). On average, *indica* rice exhibits a larger tiller angle (11.7 ± 5.8° in Hainan; 10.5 ± 5.6°in Wuhan) than *japonica* rice (8.8 ± 3.6° in Hainan; 9.1 ± 3.6° in Wuhan), and the variation within *indica* is greater than in *japonica*. The correlation coefficients between Hainan and Wuhan were 0.71 and 0.49 within the *indica* and *japonica* subpopulations, respectively. Two-way analysis of variance (ANOVA) revealed that tiller angle was dominantly controlled by genetic factors but was also influenced by interactions between genotype and environment (Table 1). In the *japonica* subpopulation in particular, the interaction between genotype and environment accounted for 22.6% of the variation in tiller angle (Table 1). Tiller angle had a high heritability of 0.82.

**Table 1:** Summary of variances resolved by two-way ANOVA for rice tiller angle in two environments.

SOV	Full population				*Indica* subpopulation				*Japonica* subpopulation			
	df	F	P	SSG/SST (%)	df	F	P	SSG/SST (%)	df	F	P	SSG/SST (%)
G	498	26.7	<0.0001	79.0	288	31.8	<0.0001	80.7	141	12.5	<0.0001	66.4
E	1	75.0	<0.0001	0.4	1	123.8	<0.0001	1.1	1	6.9	0.00885	0.3
G*E	498	4.9	<0.0001	14.6	288	5.2	<0.0001	13.1	141	4.2	<0.0001	22.6
Error	998				578				284			

SOV, source of variance; G, genotype; E, environment; G×E, the interaction between genotype and environment. SSG, genotype sum-of-squares; SST, total sum-of-squares.

### QTLs commonly identified in the two environments

We performed GWAS separately in the whole population and in the *indica* and *japonica* subpopulations for each year. Manhattan plots and quantile-quantile plots of the rice tiller angles among the three populations are illustrated with the results obtained from both the linear mixture method (LMM) (S1 Fig) and linear regression (LR) approaches (S2 Fig). A total of 30 tiller angle-related QTLs were detected (Table 2 and S1 Table and S2 Table). Of them, seven were commonly detected in Wuhan and Hainan. Three QTLs (*qTA1b*, *3* and *7a*) were only identified through LR, while the remaining was identified through both LMM and LR (Table 2). Among these seven QTLs, 3 (*qTA1b*, *7a* and *8b*) were detected only in the whole population, 2 (*qTA3* and *7b*) were identified only in the *indica* subpopulation, and two QTLs (*qTA8a* and *9c*) were commonly detected in the whole population and the *indica* subpopulation. No significant association signals were commonly detected in the *japonica* subpopulation in the two environments (Table 2). Two QTLs (*qTA1b* and *3*) on chromosomes 1 and 3 individually explained more than approximately 15% of the variation in the whole population and in the *indica* subpopulation, respectively. Two QTLs (*qTA8a* and *8b*) on chromosome 8 presented different contributions to the tiller angle in the two environments. The QTL of *qTA8a* exhibited the major effect in the *indica* subpopulation.

**Table 2:** Significant association loci for rice tiller angle detected in both Hainan and Wuhan using the LMM and LR.

QTLs	Pop	Chr	Local LD region (bp)	Hainan			Wuhan			Known genes
				SNP ID	P value	Var %	SNP ID	P value	Var %	
qTA1b*	All	1	4,986,351~5,464,130	sf0105241388	1.3E-25	20.5	sf0105254047	3.3E-27	16.8	*D2*
qTA3*	Ind	3	29,504,013~29,791,496	sf0329582676	3.9E-16	14.9	sf0329582676	1.1E-23	29.9	
qTA7a*	All	7	1,838,183~2,345,960	sf0701966037	6.0E-28	1.6	sf0701968196	1.8E-25	1.4	
qTA7b**	Ind	7	2,346,337~2,691,595	sf0702613618	7.2E-10	5.9	sf0702639895	9.5E-07	6.5	
qTA8a**	All	8	20,152,984~21,159,330	sf0820847844	1.1E-08	1.9	sf0820873192	9.0E-10	1.0	
qTA8a**	Ind	8	20,345,270~21,138,077	sf0820873192	5.3E-07	5.2	sf0820873192	7.1E-12	6.6	
qTA8b**	All	8	21,239,555~21,280,570	sf0821240555	4.1E-06	2.8	sf0821240555	5.9E-07	1.5	
*qTA9c*	Ind	9	20,713,333~20,883,989	sf0920743785	1.8E-08	4.1	sf0920721207	2.5E-06	2.2	*TAC1*
qTA9c**	All	9	20,713,969~20,838,372	sf0920735688	4.2E-14	5.2	sf0920728309	1.2E-07	0.6	*TAC1*

* and ** detected only by LR, both LMM and LR, respectively. Other detected only by LMM.

The SNP ID is composed of three parts: sf, the number of chromosome and the genome position (MSU.V6), eg. sf0105241388 indicates the SNP located in 5,241,388 bp on chromosome 1 (MSU.V6).

### Associations uniquely identified in one environment

In addition to these 7 QTLs commonly detected in both environments, 10 and 13 QTLs were specifically detected in Hainan (S1 Table) and Wuhan (S2 Table), respectively. In Hainan, 5, 4 and 3 QTLs were detected in the whole population and in the *indica* and *japonica* subpopulations, respectively. Two QTLs (*qTA7d* and *qTA12a*) were commonly detected in the whole population and in the *indica* subpopulation. Three QTLs were located on chromosome 7. In Wuhan, 4, 3 and 6 QTLs were detected in the whole population and in the *indica* and *japonica* subpopulations, respectively. However, no QTLs were commonly detected in either population. There were 3 QTLs on each of chromosomes 1 and 7.

### Co-localization of associated sites with previously reported QTLs for the tiller angle

In the past decade, ~11 genes were reported to control tiller angle in rice [32]. However, in the present study, only *TAC1* was detected in the local LD region via GWAS in both Hainan and Wuhan (Table 2). To evaluate the results of GWAS, we compared the localization of associated sites with those 11 tiller angle QTLs detected in cultivated rice from the gramene web site (http://www.gramene.org) and 14 significant tiller angle loci detected via GWAS in the previous study[30]. A total of 7 associated sites were found to co-localize with 6 previously reported QTLs (S3 Table). Of them, four QTLs were commonly identified in both environments. *qTA1b* and *qTA9c* were found in the regions of *QTa1* and *qTA-9a*, respectively; Both *qTA8a* and *qTA8b* were located in a large QTL interval covered a 4-Mb genome region [8, 30]. *qTA4* and *qTA7h* detected only in Wuhan and *qTA9b* detected in Hainan were identified in QTL regions. The co-localization of QTLs detected through linkage analysis and GWAS indicated their roles in controlling tiller angle.

### *TAC3*, the candidate gene of *qTA3*, controls tiller angle

**Fig 2:**
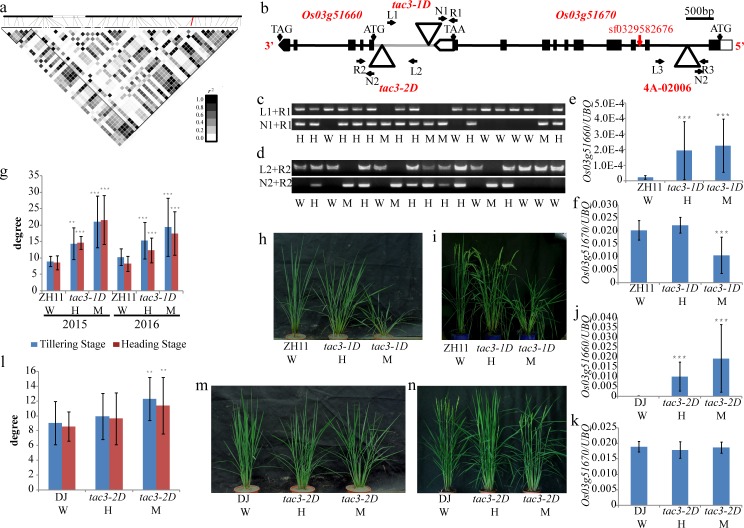
Mutants of the candidate gene for *qTA3* showing a larger tiller angle. (a) A representation of pairwise *r^2^* values (a measure of LD) all polymorphic sites between *Os03g51660* and *Os03g51670*, where the darkness of the color of each box corresponds to the *r^2^* value according to the legend. The line in red color represents lead SNP. (b) Schematic diagram of *Os03g51660* and *Os03g51670* structures with the T-DNA insertion. The black squares represent exons, and the black lines represent introns. The red line means the position of the lead SNP of *qTA3*; the triangles indicate the positions of T-DNA insertion in three mutants; L and R, left and right genomic primers; N, vector primer. (c) (d)Genotyping of the mutants *tac3-1D* and *tac3-2D*. W, wild type; H, heterozygote; M, homozygote. (e) qRT-PCR expression analysis of *Os03g51660* in wild type (ZH11) and *tac3-1D* mutant using leaves on the beginning of tillering stage; the number of plants in each genotype (n) ≥ 9, *** significant difference between mutants and wild type at p<0.001. (f) qRT-PCR expression analysis of *Os03g51670* in ZH11 and *tac3-1D* mutant using leaves on the beginning of tillering stage; the number of plants in each genotype (n) ≥4 plants, *** significant difference between mutants and wild type at p<0.001. (g)-(i) The tiller angle of ZH11, heterozygote (*tac3-1D* H) and homozygote (*tac3-1D* M) mutant at tillering and heading stages; ** and *** significant difference between mutants and wild type at p<0.01 and p<0.001, respectively. (j) qRT-PCR expression analysis of *Os03g51660* in wild type (DJ) and *tac3-2D* mutant using leaves on the beginning of tillering stage; the number of plants in each genotype (n) ≥4 plants, *** significant difference between mutants and wild type at p<0.001. (k) qRT-PCR expression analysis of *Os03g51670* in DJ and *tac3-2D* mutant using leaves on the beginning of tillering stage; the number of plants in each genotype (n) ≥4 plants. (l)-(n) The tiller angle of DJ, heterozygote (*tac3-2D* H) and homozygote (*tac3-2D* M) mutant at tillering and heading stages; the total plant number (n) = 62 plants, ** significant difference between mutants and wild type at p<0.01.

The lead SNP sf0329582676 of *qTA3* on chromosome 3, which was specifically identified in *indica* rice, is located in the second intron of *Os03g51670*, 5 kb upstream of *Os03g51660* (Fig 2b). We quantified all 49 SNPs between *Os03g51660* and *Os03g51670* in 295 *indica* accessions, and a representation of the obtained pairwise *r*^*2*^ values showed that some SNPs in both genes were in high linkage disequilibrium (LD) with each other. SNPs in the 5’ end of *Os03g51660* and *Os03g51670* were grouped into one LD block, which made it difficult to determine the candidate gene of *qTA3* (Fig 2a). A mutant (05Z11AZ62) was subsequently obtained in which a T-DNA was inserted in the 3’UTR (441 bp from stop codon) of *Os03g51670* and the promoter (991 bp from start codon) of *Os03g51660* (Fig 2b and 2c); this mutant showed a larger tiller angle compared with the wild type at both tillering and heading stages in 2015 and 2016 of Wuhan (Fig 2g–2i). A segregation analysis of a family of 96 plants (chi square = 3.56) indicated that a single gene controlled the tiller angle. The average tiller angle of homozygous mutants was the largest, while that of heterozygous mutants was intermediate, and wild type plants possessed the smallest tiller angle at both stages in two years (Fig 2g). An expression profiling analysis indicated that *Os03g51660* was highly expressed in the tiller base, and the constitutive expression of *Os03g51670* (S3d and S3e Fig) implied that *Os03g51660* was the candidate gene controlling the tiller angle. We investigated the expression level of *Os03g51670* and *Os03g51660* between mutants and wild type. The expression level of *Os03g51670* was significantly reduced only in the homozygous mutant and not in the heterozygote compared with wild type (Fig 2f). However, the expression level of *Os03g51660* was significantly increased in the homozygous mutants and the heterozygotes compared with wild type (Fig 2e). That is, the *Os03g51660* expression level co-segregated with the tiller angle. In addition, *Os03g51660* was preferentially expressed in the tiller base, where the tiller bud is initiated and outgrown. Therefore, *Os03g51660* is the gene underlying *qTA3*. Hereafter, this gene is referred to as *TAC3*, and this mutant is named as *tac3-1D*. To provide strong evidence for our results, another two mutants 1B-24636 (*tac3-2D*) with a T-DNA insertion in the 5’UTR (146 bp from start codon) of *Os03g51660* and 4A-02006 with a T-DNA insertion in the first intron of *Os03g51670* (Fig 2b) were investigated in 2016. The expression level of *Os03g51660* in both homozygous and heterozygous *tac3-2D* mutants significantly increased compared with that in wild type Dongjing (Fig 2j). The average tiller angle was no significant difference between heterozygous *tac3-2D* and wild type Dongjing at both stages, but the tiller angle of homozygous mutant (12.3°±2.9° tillering stage and 11.4°±3.8° heading stage) was significantly larger up to 3° than DJ (9.0°±2.9° tillering stage and 8.6°±2.0° heading stage) (Fig 2l–2n). Whereas the expression of *Os03g51670* was not changed in *tac3-2D* as compared to wild type (Fig 2k). Although expression of *Os03g51670* was significantly decreased in both homozygous and heterozygous 4A-02006 mutants (S3a and S3b Fig), tiller angle of mutants was not significantly changed (S3c Fig). Therefore *Os03g51660* is the identity of *TAC3* controlling tiller angle.

### Characterization of *TAC3*

The genomic DNA sequence of *TAC3* is 1,717 bp in length, with five exons and four introns (Fig 2b). Its coding sequence is 459 bp in length (S3f Fig), and it encodes a conserved hypothetical protein of 152 amino acids (http://rice.plantbiology.msu.edu/).

We detected 3 major haplotypes based on 10 SNPs (Minor Allele Frequency (MAF) ≥ 0.05) in *TAC3* among the 295 *indica* accessions (Table 3). The tiller angle of Hap1 was significantly wider than that of Hap2 and Hap3 in both environments (p≤0.01). However, only 1 G/T SNP (sf0329577726) was found in the *japonica* subpopulation, and all but two accessions carried the ‘T’ allele, which indicated that a major allele dominated in *japonica*; by contrast, the ‘G’ allele was present in all but 10 *indica* accessions. Nucleotide diversity analysis showed that both *TAC3* (π = 1.9e-5) and its surrounding genomic region (π = 1.1e-4) of 100 kb upstream and downstream presented significantly decreased values compared with the average nucleotide diversity across the whole *japonica* genome (π = 1.45e-3), indicating that *TAC3* was selected during *japonica* domestication and genetic improvement (Table 4).

**Table 3:** Comparison of genetic effects among *qTA3*/*TAC3* haplotypes on tiller angle in *indica* subpopulation.

Haplotypes	sf0329576190	sf0329576332	sf0329576631	sf0329576643	sf0329576796	sf0329577363	sf0329577374	sf0329577541	sf0329577613	sf0329577698	No. access	Tiller angle (°)	
												Hainan	Wuhan
Hap1	A	T	G	T	T	T	T	A	G	G	121	14.0±5.9A	13.3±5.7A
Hap2	C	T	C	A	C	G	T	G	G	G	79	10.1±4.7B	8.5±4.6B
Hap3	A	C	G	A	C	G	C	G	T	A	57	9.2±4.4B	8.0±3.7B

The P value of Duncan test is less than 0.01.

**Table 4:** The nucleotide diversity (π = 10^−3^) of the genes and their surrounding 100-kb regions in *indica*, *japonica* and wild rice.

Pop	WG	*qTA3/TAC3*	*TAC1*	*D2*	The regions of targeted genes		
					*qTA3/TAC3*	*TAC1*	*D2*
*Jap*	1.45	0.02	0.09	0.16	0.11	0.17	0.31
*Ind*	2.31	2.48	1.18	1.51	2.84	3.02	2.09
Wild	3.96	1.37	1.45	1.99	3.27	3.29	3.08

Pop population; WG whole genome; *Jap Japonica*, *Ind Indica*.

### *TAC1*, the candidate gene of *qTA9c*

**Fig 3:**
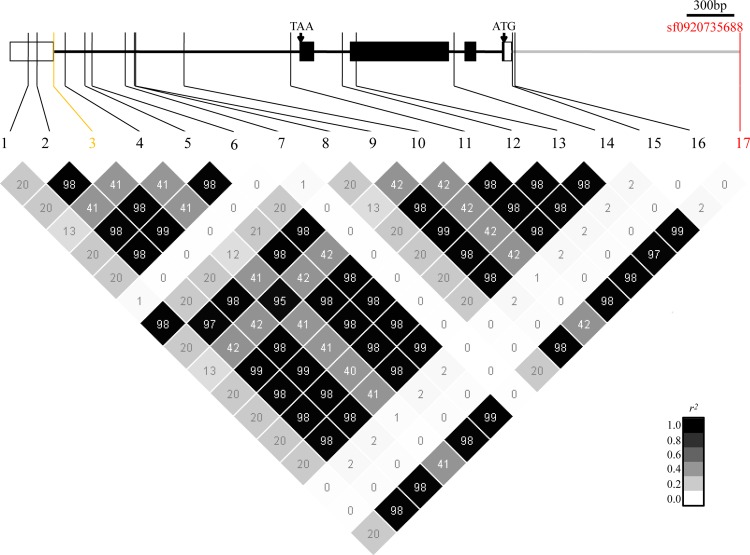
Schematic diagram of *TAC1* structure and linkage disequilibrium (measured as pairwise *r^2^* values) between the lead SNP of *qTA9c* and all polymorphic sites in *TAC1*. The white box and black box in the gene represent UTR and exon region. The darkness of each box corresponds to the *r^2^* value according to the legend. The SNP ID in yellow and red represent FNP and lead SNP respectively.

*qTA9c* was closely linked to *TAC1*, a previously reported rice tiller angle-related gene [11], and its lead SNP was located 1.4 kb upstream to *TAC1*. Assessment of the LD between the lead SNPs and all polymorphic sites in *TAC1* showed that the lead SNP sf0920735688 (P_LMM_ = 4.2e-14) was in high linkage disequilibrium with most polymorphic sites in *TAC1* containing GGGA/AGGA (sf0920731363) sites, which are known functional nucleotide polymorphic sites (FNPs) of *TAC1* (Fig 3). Therefore, *TAC1* was assumed to be the candidate gene of *qTA9c*. We further investigated a total of 13 SNPs (MAF≥0.05) throughout *TAC1* among 529 *O*. *sativa* accessions, and 16 haplotypes were detected. The vast majority (285 *indica* and 156 *japonica* accessions) of the accessions belonged to Hap1, Hap2 and Hap3 (Table 5). All but one of the *japonica* accessions were classified into Hap3 with the ‘GGGA’ allele. Most of the *indica* accessions fell into Hap1 and Hap2, which both contain the ‘AGGA’ allele, whereas 35 *indica* accessions carried Hap3. There was a highly significant difference (p<0.01) in the tiller angle between Hap1 and Hap3 in the *indica* subpopulation in both environments (Table 5). These results explained why *TAC1* was detected in the whole population and the *indica* subpopulation, but not in the *japonica* subpopulation. The nucleotide diversity of *TAC1* and its flanking region was subsequently analyzed. Accordingly, a significant reduction in nucleotide diversity was observed at *TAC1* in the *japonica* subpopulation (π = 9.4e-5) compared with both wild rice (π = 1.45e-3) and the average diversity of the whole *japonica* genome (π = 1.45e-3) (Table 4). We also observed a lower level of nucleotide diversity (π = 1.7e-4) in the 100-kb region surrounding *TAC1* in the *japonica* subpopulation compared with the whole *japonica* genome. However, no obvious changes were found in the *indica* subpopulation (π = 3.0e-3).

**Table 5:** Comparison of genetic effect among *TAC1* haplotypes on tiller angle in *indica* and *japonica* subpopulations.

Haplotypes	sf0920731204	sf0920731260	sf0920731363	sf0920731434	sf0920731559	sf0920731592	sf0920731858	sf0920731863	sf0920732165	sf0920732836	sf0920733162	sf0920733249	sf0920733864	No. *ind*	No. *jap*	Tiller angle (°)			
																Hainan		Wuhan	
																*Indica*	*Japonica*	*Indica*	*Japonica*
Hap1	G	T	**A**	T	T	G	A	T	C	G	A	G	C	163	0	13.0±5.7A		11.6±5.6A	
Hap2	T	T	**A**	G	T	G	G	T	T	G	A	G	C	87	1	10.7±5.7AB		9.3±5.7AB	
Hap3	G	G	**G**	G	C	A	A	C	T	A	T	A	T	35	155	8.3±4.1B	8.8±3.6	8.9±4.3B	9.1±3.6

The SNP in bold is FNP for *TAC1*. The P value of Duncan test is less than 0.01.

### *D2*, the candidate gene of *qTA1b*

**Fig 4:**
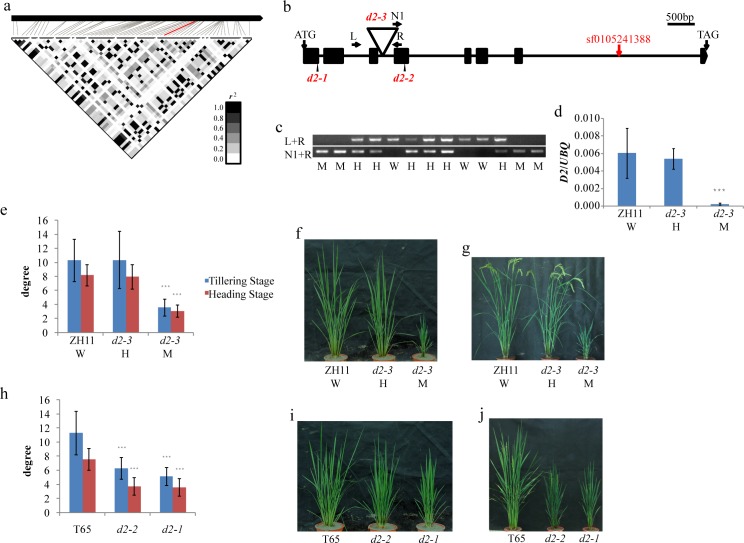
The mutant of *D2*, the candidate gene of *qTA1b*, showing decreased tiller angle. (a) A representation of pairwise *r^2^* values (a measure of LD) all polymorphic sites in *D2*, where the darkness of the color of each box corresponds to the *r^2^* value according to the legend. The line in red color represent lead SNP. (b) Schematic diagram of *D2* gene structure with the T-DNA insertion. The black squares represent exons, and the black lines represent introns. The red arrow indicates the position of lead SNP of *qTA1b*; the triangle indicates the position of T-DNA insertion; L and R, left and right genomic primers; N, vector primer. (c) genotyping of the *d2-3* mutant. W, the wild type; H, heterozygote; M, homozygote. (d) qRT-PCR expression analysis of *D2* in wild type ZH11, heterozygote (*d2-3* H)and homozygote (*d2-3* M) mutant using the tissues aboveground at the seedling stage; the number of plants in each genotype (n)≥3 plants, *** significant difference between mutants and wild type at p<0.001.(e)-(g) Phenotypes of ZH11 and *d2-3* mutant (right) at tillering stage and heading stage; the total plant number (n) = 96 plants, *** significant difference between mutants and wild type at p<0.001 (h)-(j) Phenotypes of T65, *d2-1* and *d2-2* mutant at tillering stage and heading stage; the number of plants in each genotype (n)≥15 plants, *** significant difference between mutants and wild type at p<0.001.

Brassinosteroid, as one of the main plant hormones, is considered to be an important factor in plant development, including the tiller angle. The lead SNP sf0105241388 of *qTA1b* is located in the last intron of *DWARF 2* (*D2*). A representation of the obtained pairwise *r*^*2*^ values showed that all SNPs in *D2* were in one LD block (Fig 4a). The *d2-1* and *d2-2* mutant shows a brassinosteroid-deficient phenotype including an erect leaf angle and a short plant height, but the tiller angle of this mutant was not mentioned [33]. Thus, the tiller angle of a mutant we named *d2-3* (04Z11MY27) with a T-DNA insertion in the third intron of *D2* was investigated (Fig 4b). Genotyping (Fig 4c) and an expression analysis (Fig 4d) suggested that the *d2-3* mutant exhibited abnormal growth, including a smaller tiller angle (Fig 4e–4g). This result suggested *D2* as the candidate gene of *qTA1b*. We further investigated the tiller angle of *d2-1* and *d2-2* to solid our study, and found the tiller angles of both mutants (*d2-1*: 5.2°±1.3° tillering stage and 3.6°±1.2° heading stage; *d2-2*: 6.3°±1.5° tillering stage and 3.8°±1.2° heading stage) were significantly decreased as compared with that in wild type T65 (11.3°±3.1° tillering stage and 7.6°±1.5° heading stage) (Fig 4h–4j).

To better understand the natural variation of *D2*, we further analyzed its haplotypes. We constructed the haplotypes of *D2* within two subpopulations based on the SNPs with an MAF ≥ 0.05. In the *japonica* subpopulation, we obtained four main haplotypes excluding the lead SNP sf0105241388 because all but one accession carried the same allele, ‘A’, at this site. Most of the accessions (112) fell into one haplotype. Only 13, 11 and 10 accessions fell into the other three haplotypes. The tiller angle showed no significant differences among these haplotypes (Table 6). In the *indica* subpopulation, we also obtained four main haplotypes that were different from those in the *japonica* subpopulation (Table 7). Hap1 and Hap4 exhibited significantly smaller tiller angles compared with Hap2 and Hap3 (p≤0.01). Hap1 and Hap4 carried the allele ‘A’ at the lead SNP site, whereas Hap2 and Hap3 carried the ‘G’ allele. Significantly decreased nucleotide diversity (π = 1.6e-4) was observed only in the *japonica* subpopulation compared with the average nucleotide diversity of its whole genome (Table 4).

**Table 6:** Comparison of genetic effects among *D2* haplotypes on tiller angle in *japonica* subpopulation.

Haplotypes	sf0105238516	sf0105239482	sf0105240106	sf0105240466	sf0105242183	No. *jap*	Tiller angle (°)	
							2013 Hainan	2014 Wuhan
Hap1	C	G	T	G	C	112	9.0±3.7A	9.1±3.8A
Hap2	C	A	C	G	C	13	7.7±2.9A	10.8±3.2A
Hap3	T	G	T	G	T	11	8.3±2.7A	9.5±2.9A
Hap4	C	G	T	A	C	10 (8)		

The P value of Duncan test is less than 0.01. Tiller angles of only 8 accessions in Hap4 group were measured and then Hap4 was not used for comparative analysis due to limited sample size.

**Table 7:** Comparison of genetic effects among *D2* haplotypes on tiller angle in *indica* subpopulation.

Haplotypes	sf0105236486	sf0105236810	sf0105237880	sf0105237945	sf0105238193	sf0105238592	sf0105239226	sf0105239578	sf0105239664	sf0105239748	sf0105239805	sf0105239836	sf0105240264	sf0105240302	sf0105241111	sf0105241114	sf0105241286	sf0105241388	sf0105241431	sf0105241461	sf0105241462	sf0105241540	sf0105241656	sf0105242003	sf0105242138	sf0105242226	sf0105242372	sf0105242682	sf0105242719	sf0105243076	No. *ind*	Tiller angle (°)	
																																Hainan	Wuhan
Hap1	T	G	G	C	G	A	G	C	T	T	C	C	G	C	T	A	G	**A**	G	A	A	A	G	A	G	A	T	C	G	T	125	10.1±4.8A	8.3±4.4A
Hap2	N	T	A	G	A	A	G	T	C	T	A	A	A	T	A	G	A	**G**	A	G	A	G	A	A	A	G	C	T	C	G	65	14.0±6.6B	13.3±5.9B
Hap3	T	G	G	C	A	G	A	C	T	C	C	C	G	C	T	G	A	**G**	G	G	G	G	A	G	A	G	T	C	C	G	59	14.4±6.1B	13.7±5.7B
Hap4	A	G	G	C	G	A	G	C	T	T	C	C	G	C	T	A	G	**A**	G	A	A	A	G	A	G	A	T	C	G	T	19	8.2±2.7A	6.5±1.8A

The SNP in bold is the lead SNP of *qTA1b*. The P value of Duncan test is less than 0.01.

### The effect of three-gene combinations on the tiller angle

According to the results described above, it is clear that *TAC3*, *D2* and *TAC1* together, along with other genes, contribute to the natural variation in tiller angle in cultivated rice. Thus, the combinations of *TAC3*, *D2* and *TAC1* would be expected to result in wide variation of tiller angles in cultivars.

A total of 77 combinations of the genes *TAC3*, *D2* and *TAC1* were observed in the 295 *indica* accessions, but only 9 combinations that were each found in more than 10 accessions were used for the comparative analysis (Table 8). In Wuhan and Hainan, combinations 1 and 2 both pyramided three haplotypes that increased the tiller angle, resulting in the largest tiller angle. Combinations 3–7 with decreased tiller angle haplotypes of *D2* and *TAC3*, independent of *TAC1* showed similar compact plant architecture in both environments. Combination 8 pyramided increasing tiller angle haplotypes of *D2* and *TAC3*, but a decreased tiller angle haplotype of *TAC1* resulted in a large tiller angle. Combination 9 carried increasing tiller angle haplotypes of *TAC1* and *TAC3* but a decreasing tiller angle haplotype of *D2*, and accordingly exhibited a large tiller angle as well. These results indicated that *D2* and *TAC3* were the major QTLs in the *indica* cultivars. Among all 295 *indica* accessions, 5 carried two combinations with decreasing tiller angle haplotypes at three gene loci. Accessions W066 and W155 (*TAC3-Hap3*/*D2-Hap1*/*TAC1-Hap3*) showed more compact tiller angles (W066: 3.8° in Hainan and 3.5° in Wuhan; W155: 6.0° in Hainan and 5.5° in Wuhan). Similarly, accessions W161, W243 and W244 (*TAC3-Hap2*/*D2*-*Hap1*/*TAC1-Hap3*) also presented smaller tiller angles (5.2–6.7° in Hainan and 5.2–5.7° in Wuhan).

**Table 8:** The performance of tiller angle for three-gene combinations in *indica* subpopulation.

Combinations	Haplotypes			No of accessions	Tiller angle (°)	
	*D2*	*qTA3*/*TAC3*	*TAC1*		Hainan	Wuhan
1	Hap3 ^L^	Hap1 ^L^	Hap1 ^L^	35	15.4±5.7A	14.0±5.1A
2	Hap2 ^L^	Hap1 ^L^	Hap1 ^L^	28	14.4±5.6AB	12.9±5.9A
3	Hap1 ^S^	Hap2 ^S^	Hap1 ^L^	26	10.2±4.9BC	8.6±4.3BC
4	Hap1 ^S^	Hap2 ^S^	Hap2	19	9.1±4.0C	6.6±2.3C
5	Hap1 ^S^	Hap3 ^S^	Hap1 ^L^	18	9.5±3.1C	8.2±3.9BC
6	Hap1 ^S^	Hap3 ^S^	Hap2	15	8.1±2.4C	6.9±3.2C
7	Hap4 ^S^	Hap2 ^S^	Hap2	12	7.8±2.6C	6.1±1.8C
8	Hap2 ^L^	Hap1 ^L^	Hap3 ^S^	12	11.2±5.1ABC	11.4±3.6AB
9	Hap1 ^S^	Hap1 ^L^	Hap1 ^L^	11	15.0±6.5A	10.4±6.1ABC

The haplotypes symbolized by L or S indicates the varieties with a wide tiller angle or a compact tiller angle, respectively. The others represent the varieties with a middle level tiller angle.

The P value of Duncan test is less than 0.01.

## Discussion

### Diverse genetic basis of the tiller angle between the *indica* and *japonica* subpopulations

In general, *indica* rice exhibits a wide plant type, and *japonica* rice exhibits a compact plant type. In this study, the *indica* subpopulation presented wider variation in tiller angle compared with the *japonica* subpopulation (Fig 1). Although genetic effects explained more of the variation in tiller angle (Table 1), Genotype by environment interactions also significantly affected tiller angle. Moreover, genetic factors contributed more to the variation in tiller angle in *indica* than in *japonica*. These results indicated that there are probably different genetic bases underlying the tiller angle in the two subpopulations. Coincidently, GWAS identified more QTLs for the tiller angle in the *indica* subpopulation than in the *japonica* subpopulation in both environments. This result indicates that the greater number of QTLs controlling the tiller angle in *indica* rice would contribute to wider natural variation.

Although a dozen QTLs were identified in each subpopulation, there were no common QTLs detected in both subpopulations in this study. Two QTLs, *qTA8a* and *qTA9c*, were found in both the whole population and the *indica* subpopulation, but not in the *japonica* subpopulation. It is likely that both QTLs were fixed for one major haplotype in the *japonica* accessions, resulting in failed detection. For example, the locus *qTA9c*/*TAC1* was not identified in *japonica* because *TAC1* is dominantly fixed with Hap3 in this subpopulation (Table 5). The tiller angle associated with Hap3 was significantly reduced compared with that of Hap1, the major haplotype in *indica*, which explains the compact plant architecture of *japonica* rice. There was only one SNP observed in *qTA3*/*TAC3* in *japonica*, and all but two accessions carried the dominant allele. Thus, the *japonica* accessions were almost fixed with one haplotype for *TAC3*. Accordingly, the haplotype analysis of the full population suggested the existence of *indica*-*japonica* differentiation for *D2*: no significant differences in tiller angle were observed among the four main haplotypes within the *japonica* subpopulation (Table 6), while there was a significant difference in the *indica* subpopulation (p≤0.01) (Table 7). This suggested that the major QTLs are fixed in *japonica*. Two QTLs, *qTA7a* and *qTA8b*, were only identified in the whole population and were not identified in either the *indica* or *japonica* subpopulation. A possible explanation for this finding is that the gene was fixed with different haplotypes in the two subpopulations. When GWAS was performed in each subpopulation, the QTLs were not detected because no polymorphism was present in sequence; however, when GWAS was conducted in the whole population, these QTLs were identified because variation occurred. Hence, it is recommended that GWAS should be performed separately in different populations to discover more QTLs for tiller angle or other traits. According to the above results, we propose that there is diverse genetic basis controlling the tiller angle between the two subpopulations.

### *TAC1*, *TAC3* and other genes, regulate tiller angle in rice cultivars rather than *PROG1* and *LA1*

Tiller angle is a domestication-related trait in rice, and selective signatures of domestication include a reduction of nucleotide diversity and altered allele frequencies at domestication-related loci [34,35]. The functional SNPs of *PROG1*, a gene controlling prostrate growth, only exist in wild rice [9,10]. In addition, *PROG1* was detected via GWAS in 446 *O*. *rufipogon* accessions and was screened for strong selection signals [36]. In this study, *PROG1* was not identified in the examined cultivars. *LA1* is another major gene isolated from a mutant that controls the tiller angle, and loss of function of *LA1* destroys rice shoot gravitropsim through altering the polar transport of auxin [19]. The *lazy1* mutant shows a wider tiller angle of more than 60°, especially during the mature stage, associated with a prostrate growth phenotype [18], which was never observed in our collection. Therefore, *la1* and *PROG1* do not contribute to the variation of tiller angle in our cultivars.

In this study, GWAS and a mutant analysis demonstrated that *TAC3* regulates tiller angle. The nucleotide diversity of *TAC3* in *japonica* (π = 1.9e-5) was approximately 55-fold lower than that of 111 randomly chosen gene fragments from *japonica* (π = 1.1e-3) [37], which is equivalent to the japonica whole genome estimation in our global collection (π = 1.45e-3). This result suggested that the low *TAC3* nucleotide diversity observed in *japonica* cannot be explained by a population bottleneck alone and indicated that *TAC3* was strongly selected probably due to its function in controlling tiller angle during the domestication and improvement of *japonica*. Accordingly, GWAS, a mutant analysis and nucleotide diversity analysis demonstrated that *D2* regulates the tiller angle. Thus, *TAC3* and *D2* contributed to the natural variation in the tiller angle and have been subjected to selection in *japonica*. Interestingly, the major *TAC1* haplotype in *japonica*, Hap3, was carried by only a small subset of *indica* accessions and probably introgressed from *japonica*. Our study on the selection of *TAC1* was consistent with the previous report [38]. *qTA8a* and *qTA8b* were approximately 400 kb away on chromosome 8 and were detected via both the LMM and LR approaches. Interestingly, a locus (marker seq-rs3945) associated with tiller angle at tillering stage in *indica* rice was fallen into the region of *qTA8a* and several candidate genes on this QTL were predicted by expression analysis [30]. Thus, the 400-kb region containing *qTA8a* and *qTA8b* should be further studied by developing an *indica* bi-parental mapping population. Additionally, there were several QTLs that co-localized with previously reported QTLs, while some QTLs were novel. Hence, *TAC3*, *D2*, *TAC1* and other unknown genes contribute to the tiller angle variation observed in the cultivars.

### Favorable haplotypes/alleles for the improvement of tiller angle in rice

Rice tiller angle is a major component of plant architecture that has been subject to selection from both nature and humans over a long time period. Mining of more favorable alleles of tiller angle genes is required to achieve ideal plant architecture in rice. At the single-gene level, we identified favorable haplotypes for three tiller angle genes. Hap2 (represented by Yuexiangzhan) and Hap3 (represented by 9311) of *TAC3* decrease tiller angle, as does Hap3 (represented by Zhenshan 97 in *indica* and Zhonghua 11 in *japonica*) of *TAC1*. Hap1 (represented by Minghui 63 and 9311) and Hap4 (represented by IR72) of *D2* also decrease tiller angle. A three-gene combination analysis of *D2*, *TAC3* and *TAC1* in *indica* showed that these three genes cause wide variation in the tiller angle, ranging from 5.2° to 15.4° on average, and combinations pyramiding decreased tiller angle haplotypes result in a more compact plant architecture. These results indicated that these genes function in the regulation of tiller angle and that their effects could be additive. Therefore, optimized haplotype-combinations among these three genes could serve as targets for designed breeding. More specifically, most of the *indica* cultivars carried *TAC1* haplotypes that increased tiller angle; thus, the *TAC1* haplotype Hap3 is the first option for improving plant architecture in *indica*. As most *japonica* cultivars carried compact haplotypes for *TAC1*, *TAC3* and *D2*, selecting for the haplotypes of other unknown genes that decrease the tiller angle would be the first option for improving the tiller angle in *japonica*.

In summary, the tiller angle of cultivated rice is mainly controlled by genetic factors but is also affected, to some extent, by interactions between genotype and environment. The genetic basis of tiller angle is diverse between *indica* and *japonica* rice. *TAC3*, *D2* and *TAC1* were found to be the main factors regulating tiller angle. Introgression between two subpopulations would be an efficient means of optimizing the plant architecture through designed molecular breeding.

## Materials and Methods

### Plant materials and field experiments

An association panel consisting of 529 *O*. *sativa* landraces and elite accessions was sown at the experimental farm of Huazhong Agricultural University in the winter of 2013 in Hainan and in the 2014 rice growing season in Wuhan (China). The 2-year field experiment was designed with 2 replicates per year. Seven 25-day-old seedlings from these accessions were transplanted in a single row with a distance of 16.5 cm between plants and 26.4 cm between rows on December 30, 2013 and May 12, 2014. The 5 plants in the middle were used to investigate the tiller angle at the heading stage. A protractor was employed to measure the angle between the most distant tillers on the two sides of the culm base, and half of the angle was treated as the tiller angle of the individual plant. The average tiller angle across 2 replicates within one year was used for GWAS. The SNPs of the 529 *O*. *sativa* accessions are available in the RiceVarMap (http://ricevarmap.ncpgr.cn/) [39]. The tiller angles of the 529 *O*. *sativa* accessions are listed in S4 Table.

### Two-way analysis of variance and heritability

Two-way analyses of variance were separately used to test significant difference between environments and genotypes for the whole population and two subpopulations. The analysis was run in the program Statistica 7.0 (StatSoft. Tulsa, OK, USA). Broad-sense heritability (*H*^*2*^) of rice tiller angle in the whole population was calculated based on the experiments using the formula: $H^{2}=\delta_{g}^{2}/(\delta_{g}^{2}+\delta_{ge}^{2}/n+\delta_{e}^{2}/nr)$, where $\delta_{g}^{2}$, $\delta_{e}^{2}$ and $\delta_{ge}^{2}$ were the estimates of genetic, genotype by environment and error variances derived from the mean square expectations of two-way analysis of variance (ANOVA), respectively; n was the number of environments and r was the number of replicates.

### Linkage disequilibrium and haplotype analyses

Linkage disequilibrium (LD) was investigated based on standardized disequilibrium coefficients (*D’*), and squared allele-frequency correlations (*r*^*2*^) for pairs of SNP loci were determined using the TASSEL5.0 program. The extent of genome-wide and chromosome-wide LD were recently reported [40], and the average distances of LD decay at the genome-wide level in *all*, *indica* and *japonica* populations were 167 kb, 93 kb and 171 kb, respectively. The distances of LD decay in the regions surrounding lead SNPs identified in this study were calculated as below: First, *r^2^* values were calculated between lead SNP and all SNPs in its upstream and downstream 2 Mb regions. Then averaged *r^2^* of the top ten percent of *r^2^* values in the region from 1.5 Mb to 2 Mb away from lead SNP were taken as background *r^2^*. Finally the LD region was defined a continue region where *r^2^* was 0.2 larger than background *r^2^*. LD plots were generated with Haploview4.2, and LD is indicated using *r*^*2*^ values between pairs of SNPs multiplied by 100; white, *r*^*2*^ = 0; shades of gray, 0<*r*^*2*^<1; black, *r*^*2*^ = 1 [41,42]. The SNPs of targeted genes in the 529 *O*. *sativa* samples were obtained from the RiceVarMap (http://ricevarmap.ncpgr.cn/) using the gene ID [39]. The haplotypes were classified based on all SNPs with an MAF ≥ 0.05 in a target gene. The haplotypes contains at least 10 investigated accessions were used for comparative analysis. Duncan’s test was employed to compare the differences in the tiller angle among haplotypes using the SSPE program [43].

### Genome-wide association analyses

The whole population was previously demonstrated to present a distinct population structure [31]. The *indica* and *japonica* subpopulations and the whole population were subjected to GWAS separately because they presented sample sizes of greater than one hundred. A total of 3,916,415, 2,767,159 and 1,857,845 SNPs (minor allele frequency (MAF) ≥ 0.05; the number of accessions with minor alleles ≥ 6) were employed for GWAS using the linear mixture method (LMM) and linear regression (LR) method in the FaST-LMM program [44]. The population structure of Q matrix and kinship (K matrix) was taken into account as cofactor when performing association mapping using the LMM method. The effective numbers of independent SNPs and suggestive thresholds were calculated using a method described by Li et al. [45], and 757,578, 571,843 and 245,348 effective independent SNPs were found in the whole population and the *indica* and *japonica* subpopulations, respectively. The suggestive p values used as thresholds for the significance of association signals that were commonly detected in both environments by LMM were 1.3×10^−6^ for the whole population, 1.8×10^−6^ for *indica* and 4.1×10^−6^ for *japonica*. However, for significant association signals that were only detected in one environment, we utilized the more stringent p value of 6.0×10^−7^ as the threshold. A suggestive p value of 1.0×10^−8^ was employed as the threshold for the significance of association signals detected by LR, but only the top 5 loci detected by LR in each environment are presented in the results. The loci that were commonly identified by LR in both environments, but were not in the top 5 are also presented. For loci that were commonly detected by two methods, only the results of LMM are presented. To obtain independent association signals, multiple SNPs exceeding the threshold in a 5-Mb region were clustered based on an *r^2^* of LD ≥ 0.25; the SNPs showing the minimum p value in a cluster were considered to be lead SNPs.

### Nucleotide diversity analyses

The whole genomic DNA sequences of the 529 cultivar accessions were genotyped with approximately 2.5× coverage, and the genome was sequenced using a bar-coded multiplex sequencing approach on an Illumina Genome Analyzer II [31]. We obtained the genome sequences from RiceVarMap (http://ricevarmap.ncpgr.cn/) [39]. A total of 446 *O*. *rufipogon* accessions were used to calculate nucleotide diversity (π). The details of these accessions and their sequencing data have been previously reported [36]. π values were estimated at the whole-genome level, the single gene level, and the 100-kb flanking region level using SAMtools [46].

### Genotyping of mutant plants

The *d2-3* (04Z11MY27), *tac3-1D* (05Z11AZ62), *tac3-2D* (1B-24636) and 4A-02006 mutants were obtained from rice T-DNA insertion libraries from the ZH11 variety [47,48] and DJ variety [49,50]. We identified the genotypes of the mutants via PCR using the genomic primers L and R and the vector primer N (S4 Table). PCR was conducted with an initial incubation step at 95°C for 5 min; a second step of 35 cycles at 95°C for 30 s, 58°C for 30 s, and 72°C for 1 min; and a final extension at 72°C for 7 min.

### RNA extraction and expression analysis

Total RNA was extracted from different plant tissues with an RNA extraction kit using TRIzol reagent (Invitrogen) for quantitative real-time reverse transcription-polymerase chain reaction (qRT-PCR). The total RNA (4g) was reverse-transcribed using M-MLV reverse transcriptase (Invitrogen). qRT-PCR was carried out in a total volume of 10 μl containing 2.5 μl of the reverse-transcribed products, 0.25 μM gene-specific primers and 5 μl of Fast Start Universal SYBR Green Master (Rox) superMIX (Roche, Mannheim, Germany) in a QuantStudio (TM) 6 Flex System, according to the manufacturer’s introductions. Measurements were obtained using the relative quantification method. Expression levels were normalized against expression of an *ubiquitin* (*UBQ*) gene. Error bars indicate standard deviations (n = 3). All primers employed for qRT-PCR are listed in S5 Table.

## Supporting Information

S1 Fig Genome-wide association study for tiller angle in the whole population, the *indica* and the *japonica* subpopulations by LMM. Manhattan plots and quantile-quantile plots for tiller angle in the full population (a), *indica* subpopulation (b) and *japonica* subpopulation (c). The horizontal dashed lines of the Manhattan plots indicate the significance thresholds that are defined in the section of materials and methods. Lambda of quantile-quantile plots represents the expected null distribution and the observed p value.
(TIF)

S2 Fig Genome-wide association study for tiller angle in the whole population, the *indica* and the *japonica* subpopulations by LR. Manhattan plots and quantile-quantile plot for tiller angle in the full population (a), *indica* subpopulation (b) and *japonica* subpopulation (c). The horizontal dashed lines of the Manhattan plots indicate the significance thresholds that are defined in the section of materials and methods. Lambda of quantile-quantile plots represents the expected null distribution and the observed p value.
(TIF)

S3 Fig Expression profiles of *Os03g51660* (*TAC3*) and *Os03g51670* and the sequence of *TAC3* cDNA. (a) Genotyping of the 4A-02006 mutant. W, the wild type; H, heterozygote; M, homozygote. (b) qRT-PCR expression analysis of *Os03g51670* in wild type DJ, heterozygote (4A-02006 H)and homozygote (4A-02006 M) mutant using the leaves of tillering stage; the number of plants in each genotype (n)≥3, *** p<0.001. (c) Phenotypes of wild type (n = 16), 4A-02006 H (n = 47) and 4A-02006 M (n = 21) at tillering stage and heading stage. (d), (e) Expression of *Os03g51660* (*TAC3*) and *Os03g51670*, the tissues followed by 1 and 2 were collected at tillering stage and heading stage, respectively. (f) *TAC3* cDNA is of 459 bp.
(TIF)

S1 Table Significant signals for tiller angle detected only in Hainan using the LMM and LR methods. (DOC)

S2 Table Significant signals for tiller angle detected only in Wuhan using the LMM and LR methods. (DOC)

S3 Table Co-localization of associated sites with the previously detected tiller angle-related QTLs in rice. (DOC)

S4 Table The tiller angle of 529 *O*. *sativa* in Hainan and Wuhan. (XLS)

S5 Table Primers used in this study. (DOC)
