# Supplementary figures and images for: A Novel Tiller Angle Gene, TAC3, together with TAC1 and D2 Largely Determine the Natural Variation of Tiller Angle in Rice Cultivars
Source: PLoS Genet. 2016 Nov 4;12(11):e1006412. doi: 10.1371/journal.pgen.1006412 (PMC5096673; doi:10.1371/journal.pgen.1006412)

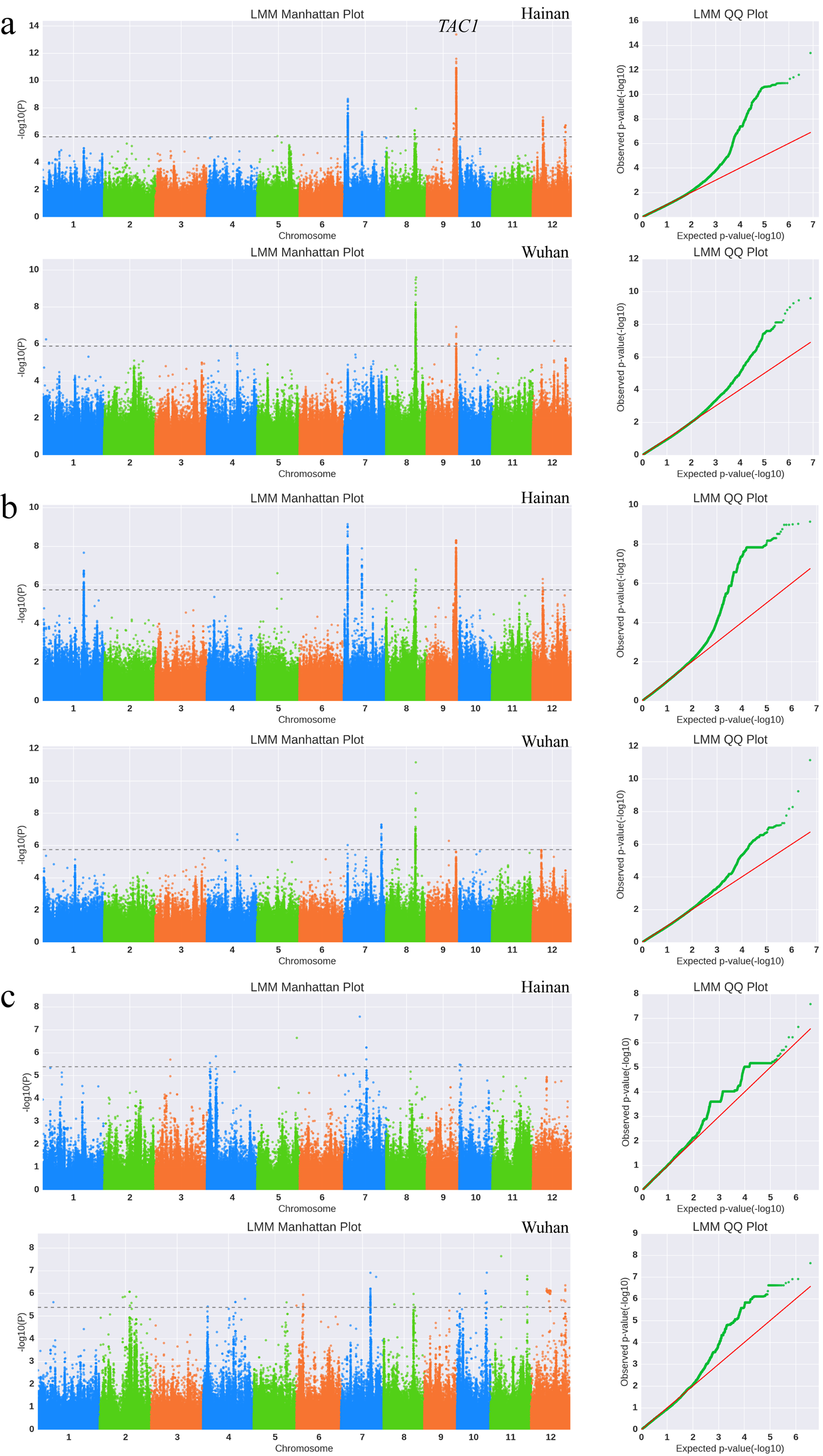

Supplement: S1 Fig — Manhattan plots and quantile-quantile plots for tiller angle in the full population (a), indica subpopulation (b) and japonica subpopulation (c). The horizontal dashed lines of the Manhattan plots indicate the significance thresholds that are defined in the section of materials and methods. Lambda of quantile-quantile plots represents the expected null distribution and the observed p value. (TIF) [file pgen.1006412.s001.tif]

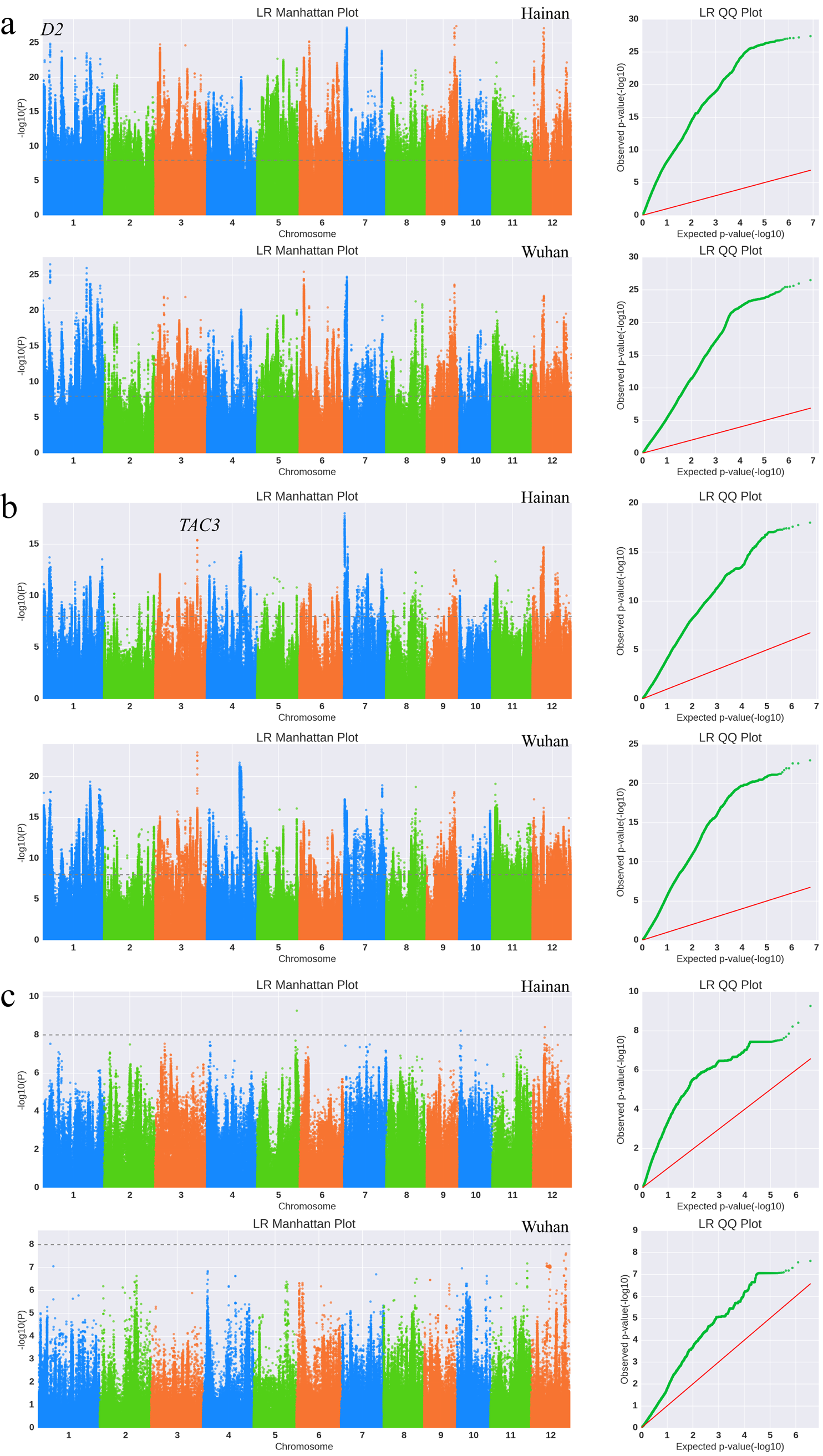

Supplement: S2 Fig — Manhattan plots and quantile-quantile plot for tiller angle in the full population (a), indica subpopulation (b) and japonica subpopulation (c). The horizontal dashed lines of the Manhattan plots indicate the significance thresholds that are defined in the section of materials and methods. Lambda of quantile-quantile plots represents the expected null distribution and the observed p value. (TIF) [file pgen.1006412.s002.tif]

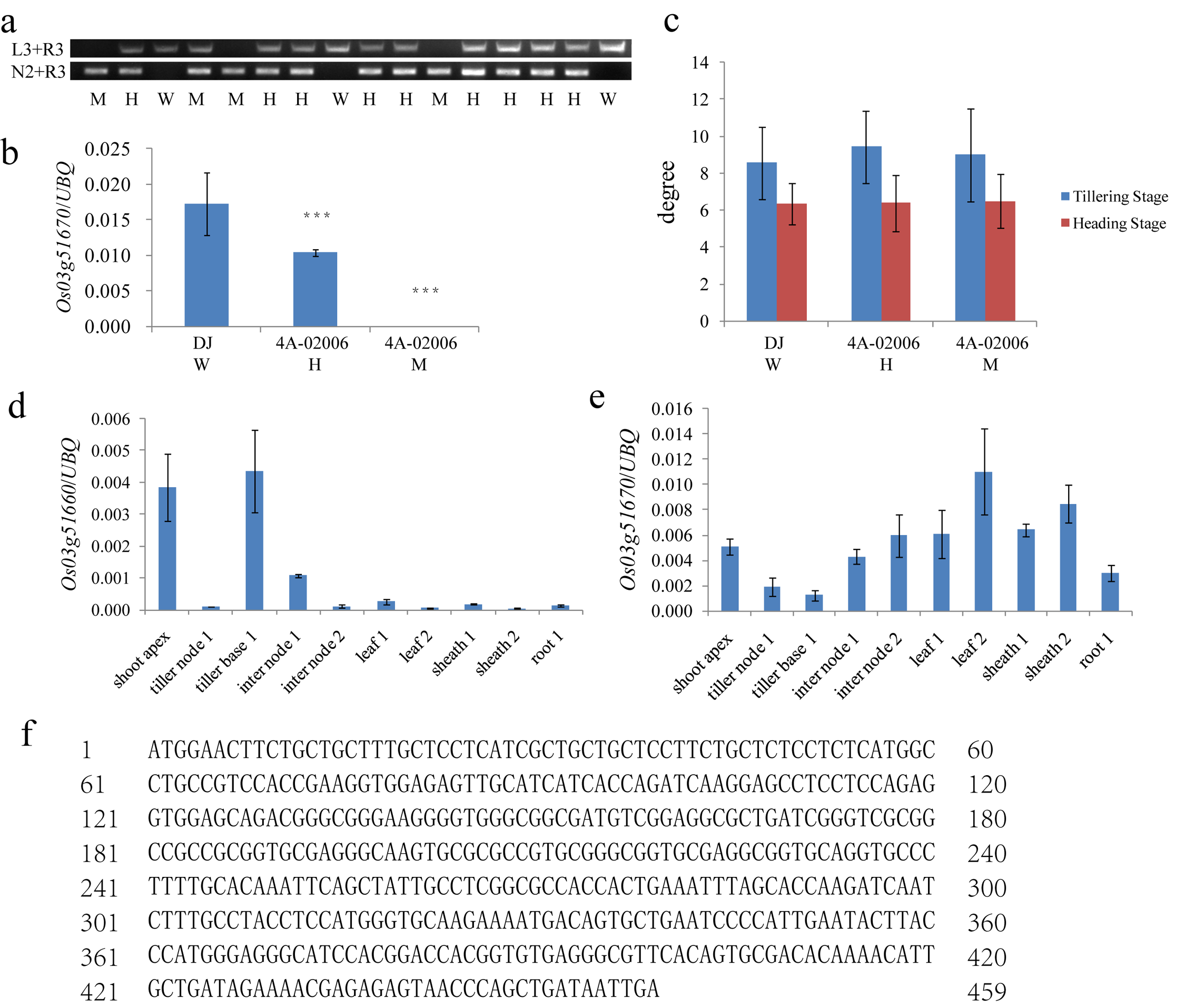

Supplement: S3 Fig — (a) Genotyping of the 4A-02006 mutant. W, the wild type; H, heterozygote; M, homozygote. (b) qRT-PCR expression analysis of Os03g51670 in wild type DJ, heterozygote (4A-02006 H)and homozygote (4A-02006 M) mutant using the leaves of tillering stage; the number of plants in each genotype (n)≥3, *** p<0.001. (c) Phenotypes of wild type (n = 16), 4A-02006 H (n = 47) and 4A-02006 M (n = 21) at tillering stage and heading stage. (d), (e) Expression of Os03g51660 (TAC3) and Os03g51670, the tissues followed by 1 and 2 were collected at tillering stage and heading stage, respectively. (f) TAC3 cDNA is of 459 bp. (TIF) [file pgen.1006412.s003.tif]
