# Supplementary material for: A Novel Tiller Angle Gene, TAC3, together with TAC1 and D2 Largely Determine the Natural Variation of Tiller Angle in Rice Cultivars
Source: PLoS Genet. 2016 Nov 4;12(11):e1006412. doi: 10.1371/journal.pgen.1006412 (PMC5096673; doi:10.1371/journal.pgen.1006412)
Supplement: S1 Table — (DOC) [file pgen.1006412.s004.doc]

**S1 Table. Significant signals for tiller angle detected only in Hainan using the LMM and LR methods.**

| QTL | Pop | Chr | Local LD region (bp) | SNP ID | P value | Var % |
| --- | --- | --- | --- | --- | --- | --- |
| *qTA1c*** | Ind | 1 | 28,763,733~29,148,969 | sf0128962797 | 2.2e-08 | 14.9 |
| *qTA5a*** | Ind | 5 | 14,247,423~14,339,622 | sf0514248423 | 2.5e-07 | 9.6 |
| *qTA5b*** | Jap | 5 | 27,864,021~27,865,021 | sf0527865021 | 2.3e-07 | 25.6 |
| *qTA6b** | All | 6 | 6,502,561~7,109,523 | sf0606678664 | 6.4e-26 | 4.9 |
| *qTA7c* | Jap | 7 | 10,895,043~11,051,286 | sf0711047800 | 2.6e-08 | 10.6 |
| *qTA7d*** | Ind | 7 | 12,625,239~12,634,443 | sf0712633357 | 1.3e-08 | 5.5 |
| *qTA7d*** | All | 7 | 12,630,698~13,291,498 | sf0712634082 | 5.7e-07 | 6.0 |
| *qTA7e* | Jap | 7 | 15,676,143~16,025,602 | sf0715678172 | 5.9e-07 | 2.3 |
| *qTA9b*** | All | 9 | 18,834,270~19,034,031 | sf0918983049 | 1.4e-07 | 1.5 |
| *qTA12a*** | All | 12 | 6,954,430~7,223,002 | sf1207176839 | 4.9e-08 | 3.0 |
| *qTA12a*** | Ind | 12 | 6,884,887~7,741,720 | sf1207183978 | 5.0e-07 | 2.2 |
| *qTA12b*** | All | 12 | 22,690,769~23,403,879 | sf1223041578 | 1.9e-07 | 1.6 |

* and ** detected only by LR, both LMM and LR methods; and others detected only by LMM.

The SNP ID is composed of three parts: sf, the number of chromosome and the genome position (MSU.V6), eg. sf0128962797 indicates the SNP located in 28,962,797 bp on chromosome 1 (MSU.V6).
