# Supplementary material for: A Novel Tiller Angle Gene, TAC3, together with TAC1 and D2 Largely Determine the Natural Variation of Tiller Angle in Rice Cultivars
Source: PLoS Genet. 2016 Nov 4;12(11):e1006412. doi: 10.1371/journal.pgen.1006412 (PMC5096673; doi:10.1371/journal.pgen.1006412)
Supplement: S2 Table — (DOC) [file pgen.1006412.s005.doc]

**S2 Table. Significant signals for tiller angle detected only in Wuhan using the LMM and LR methods.**

| QTL | Pop | chr | Local LD region (bp) | SNP ID | P value | Var % |
| --- | --- | --- | --- | --- | --- | --- |
| *qTA1a* | All | 1 | 2,310,583~2,875,680 | sf0102355976 | 5.6e-07 | 7.8 |
| *qTA1d** | All | 1 | 30,649,662~31,161,264 | sf0130928347 | 1.1e-26 | 3.5 |
| *qTA1e** | All | 1 | 33,591,439~34,535,268 | sf0133609922 | 1.8e-24 | 0.6 |
| *qTA4*** | Ind | 4 | 21,186,192~21,437,132 | sf0421249161 | 2.0e-07 | 8.1 |
| *qTA6a** | All | 6 | 2,813,580~3,272,190 | sf0602900101 | 3.7e-26 | 4.6 |
| *qTA7f* | Jap | 7 | 19,650,839~20,522,249 | sf0720482231 | 1.2e-07 | 5.1 |
| *qTA7g* | Jap | 7 | 24,464,138~24,466,138 | sf0724465138 | 1.9e-07 | 17.2 |
| *qTA7h*** | Ind | 7 | 26,283,453~26,500,242 | sf0726365945 | 5.1e-08 | 3.2 |
| *qTA9a*** | Ind | 9 | 15,555,759~15,805,910 | sf0915707204 | 5.2e-07 | 1.2 |
| *qTA10* | Jap | 10 | 20,588,973~20,781,438 | sf1020743926 | 1.2e-07 | 3.4 |
| *qTA11a* | Jap | 11 | 7,579,489~7,761,447 | sf1107664231 | 2.3e-08 | 4.7 |
| *qTA11b* | Jap | 11 | 25,855,479~25,940,854 | sf1125857792 | 1.7e-07 | 6.2 |
| *qTA12c* | Jap | 12 | 23,968,680~23,997,055 | sf1223977151 | 4.4e-07 | 0.2 |

*, ** detected only by LR, both LMM and LR methods, and others detected only by LMM.

The SNP ID is composed of three parts: sf, the number of chromosome and the genome position (MSU.V6), eg. sf0128962797 indicates the SNP located in 28,962,797 bp on chromosome 1 (MSU.V6).
