# Supplementary material for: A Novel Tiller Angle Gene, TAC3, together with TAC1 and D2 Largely Determine the Natural Variation of Tiller Angle in Rice Cultivars
Source: PLoS Genet. 2016 Nov 4;12(11):e1006412. doi: 10.1371/journal.pgen.1006412 (PMC5096673; doi:10.1371/journal.pgen.1006412)
Supplement: S3 Table — (DOC) [file pgen.1006412.s006.doc]

**S3 Table. Co-localization of associated sites with the previously detected tiller angle-related QTLs in rice.**

| QTL by GWAS | Chr | Position 1 (bp) | Previous QTLs | Marker intervals | Position 2 (bp) | Ref |
| --- | --- | --- | --- | --- | --- | --- |
| *qTA1b* | 1 | 5,241,388 | *QTa1* | RZ288~RG811 | 3,051,265~11,064,656 | [4] |
| *qTA4* | 4 | 21,249,161 | *Sdn1* | RG776~ RG449 | 17,695,908~31,452,885 | [7] |
| *qTA7h* | 7 | 26,365,945 | *ta7* | CDO497~RZ978 | 25,103,324~28,411,532 | [8] |
| *qTA8a* | 8 | 20,873,192 | *ta8*, seq-rs3945 | RG978~ RG1 | 17,437,513~21,647,445 | [8,30] |
| *qTA8b* | 8 | 21,240,555 | *ta8*, seq-rs3945 | RG978~ RG1 | 17,437,513~21,647,445 | [8,30] |
| *qTA9b* | 9 | 18,983,049 | *Ta, ta9* | RZ228~RZ12 | 18,513,777~19,427,299 | [4,8] |
| *qTA9c/ (TAC1)* | 9 | 20,735,688 | *qTA-9a*, seq-rs4356 | RG662~RM215 | 20,481,606~21,189,283 | [5,30] |

Position 1 is the genomic position (MSU.V6) of leader SNP for QTL detected by GWAS in this study.

Position 2 is the genomic region (MSU.V6) of previous QTLs.
