# Supplementary material for: A Novel Tiller Angle Gene, TAC3, together with TAC1 and D2 Largely Determine the Natural Variation of Tiller Angle in Rice Cultivars
Source: PLoS Genet. 2016 Nov 4;12(11):e1006412. doi: 10.1371/journal.pgen.1006412 (PMC5096673; doi:10.1371/journal.pgen.1006412)
Supplement: S5 Table — (DOC) [file pgen.1006412.s008.doc]

**S5 Table. Primers used in this study.**

| Name | Primer sequence(5'-3') |
| --- | --- |
| Primers for identifying mutants |  |
| 04Z11MY27 mutant |  |
| L | TGGATGAATTGGAGGAGGAG |
| R | CGATCATGTTGTCGGAGATG |
| 05Z11AZ62 mutant |  |
| L1 | CGAGTAGCTACGGATGAGGC |
| R1 | TTCTTCAACTCTGATGGGGC |
| 1B-24636 mutant |  |
| L2 | TGCAACCTGCGTGACTATTC |
| R2 | CAGGTGTTCGTTCATATGCG |
| 4A-02006 mutant |  |
| L3 | TGAAAATTGCAGCCAGATTG |
| R3 | ACCCTCTTTCGCTTTGTGTC |
| Vector primer |  |
| N1 | AATCCAGATCCCCCGAATTA |
| N2 | CTAGAGTCGAGAATTCAGTACA |
| primers for qRT-PCR analysis |  |
| *D2* |  |
| L | CACTCCTTTTGGTGGTGGGC |
| R | GGTGGGGAAGTTGACGATGTG |
| *Os03g51660* |  |
| L | CTTTGCTCCTCATCGCTGCT |
| R | AGGCTCCTTGATCTGGTGATG |
| *Os03g51670* |  |
| L | TCATAGCACCATTCAGGTCAAGA |
| R | TCCCATCCCGACCAATGTTA |
| *UBQ* |  |
| L | AACCAGCTGAGGCCCAAGA |
| R | ACGATTGATTTAACCAGTCCATGA |
